# A Selected Core Microbiome Drives the Early Stages of Three Popular Italian Cheese Manufactures

**DOI:** 10.1371/journal.pone.0089680

**Published:** 2014-02-24

**Authors:** Francesca De Filippis, Antonietta La Storia, Giuseppina Stellato, Monica Gatti, Danilo Ercolini

**Affiliations:** 1 Division of Microbiology, Department of Agricultural Sciences, University of Naples Federico II, Portici, Italy; 2 Department of Food Science, University of Parma, Parma, Italy; 3 Multidisciplinary Interdepartmental Dairy Center - MILC, University of Parma, Parma, Italy; Teagasc Food Research Centre, Ireland

## Abstract

Mozzarella (M), Grana Padano (GP) and Parmigiano Reggiano (PR) are three of the most important traditional Italian cheeses. In the three cheese manufactures the initial fermentation is carried out by adding natural whey cultures (NWCs) according to a back-slopping procedure. In this study, NWCs and the corresponding curds from M, GP and PR manufactures were analyzed by culture-independent pyrosequencing of the amplified V1–V3 regions of the 16S rRNA gene, in order to provide insights into the microbiota involved in the curd acidification. Moreover, culture-independent high-throughput sequencing of *lacS* gene amplicons was carried out to evaluate the biodiversity occurring within the *S. thermophilus* species. Beta diversity analysis showed a species-based differentiation between GP-PR and M manufactures indicating differences between the preparations. Nevertheless, all the samples shared a naturally-selected core microbiome, that is involved in the curd acidification. Type-level variability within *S. thermophilus* species was also found and twenty-eight *lacS* gene sequence types were identified. Although *lacS* gene did not prove variable enough within *S. thermophilus* species to be used for quantitative biotype monitoring, the possibility of using non rRNA targets for quantitative biotype identification in food was highlighted.

## Introduction

Mozzarella (M), Grana Padano (GP) and Parmigiano Reggiano (PR) are three of the most important traditional Italian cheeses. They are all protected designation of origin (PDO) cheeses and the technology of manufacture, as well as the microbiota involved, have been described in previous works [1]–[6]. Mozzarella is a “pasta filata” cheese traditionally produced in Southern Italy. The cheese is made from whole raw water buffalo’s milk by adding natural whey culture (NWC) as starter in a 5-h curd fermentation. PR and GP are hard, cooked cheeses made from raw, partly skimmed cow’s milk supplemented with NWC. Although completely different production technologies are employed, all these cheeses share the use of the NWC from the production of the previous day as starter for the curd acidification, according to the traditional back-slopping procedure. The microbiota of the natural starters has been characterized using both traditional and molecular procedures and defined as a consortium of micro-organisms of great importance for the quality of the traditional products. The concomitant pressure of both temperature and low pH leads to the selection of a characteristic microbiota, consisting of thermophilic, aciduric, and moderately heat resistant lactic acid bacteria (LAB), that play an important role in the achievement of the typical and appreciated sensory characteristics of cheese [7]. NWCs are generally characterized by a LAB community including both thermophilic and mesophilic bacteria [1], [3], [8]–[10]. Besides diversity at species level, the strain diversity of bacteria involved in cheese manufacture is considered a technologically important aspect [8], [11], [1] and many efforts have been done to implement reliable methods for strain discrimination and monitoring [12]–[15]. *Streptococcus thermophilus* is one of the most important bacteria in the dairy industry and it occurs in NWCs used in the manufacture of several traditional cheeses [3]–[5], [8], [16], [17]. Monitoring of this species at biotype level is an important target of the dairy industry and several molecular methods can be used [18]–[22]. The 250 bp variable region upstream from the *lacS* gene showed a high level of heterogeneity in a previous study [23], making it a good candidate for sequencing-based biotype monitoring of *S. thermophilus*.

In this study, we used culture-independent high-throughput sequencing (HTS) of 16S rRNA gene amplicons to study in depth the microbial diversity of NWCs from three Italian traditional cheeses and its evolution during curd fermentation. Moreover, a new approach was considered, to verify the possibility of using *lacS* gene amplicons pyrosequencing for *S. thermophilus* biotype monitoring.

## Materials and Methods

### Sampling

Samples from M cheese manufactures were collected from twelve dairies producing top-quality traditional water buffalo mozzarella PDO cheese, located in the Campania region (Southern Italy) in the provinces of Salerno and Caserta. Samples from GP and PR manufactures were collected from six and seven dairies located in different places within the GP and PR area of production (Northern Italy). Samples of NWCs and curds at end of the ripening were aseptically collected, cooled at 4°C, and analyzed within 6 h. NWC samples were from the manufacture of the previous day and used for the production of the corresponding curds according to the traditional back-slopping procedure. Curd samples were collected after 5 h from the adding of the NWC for M cheese and after 24 h for GP and PR cheese.

All the samples were collected and used with the permission of the dairies.

No animals were involved in the present study, but only animal products.

### DNA Extraction

Total DNA extraction from the dairy samples was carried out by using a Biostic bacteremia DNA isolation kit (Mo Bio Laboratories, Inc., Carlsbad, CA). The extraction protocol was applied to the pellet (12,000×g) obtained from 2 ml of NWC or from 2 ml of a homogenized 2-fold dilution of the curd in one-quarter-strength Ringer’s solution (Oxoid, Milano, Italy).

### 16S rRNA Gene Amplicon Library Preparation and Sequencing

The microbial diversity was studied by pyrosequencing of the amplified V1–V3 region of the 16S rRNA gene amplifying a fragment of 520 bp using primers and PCR conditions previously described [3]. 454-adaptors were included in the forward primer followed by a 10 bp sample-specific Multiplex Identifier (MID). After agarose gel electrophoresis, PCR products were purified twice by Agencourt AMPure kit (Beckman Coulter, Milano, Italy), quantified using the QuantiFluor™ (Promega, Milano, Italy) and an equimolar pool was obtained prior to further processing. The amplicon pool was used for pyrosequencing on a GS Junior platform (454 Life Sciences, Roche, Italy) according to the manufacturer’s instructions by using a Titanium chemistry.

### *lacS* Gene Amplicon Library Preparation and Sequencing

In order to prepare amplicon libraries for *S. thermophilus lacS* gene sequencing, the 454 Universal Tailed Amplicon protocol was used with a double PCR step (454 Sequencing System – Guidelines for Amplicon Experimental Design). The variable region of 250 bp upstream from the *lacS* gene was amplified using the primers LCS62f 5′- GGCTTCCAATACTTTAATT and LCS312r 5′- AAGTGAGTTGTCACAAACAT [23]. The universal primers M13f 5′-TGTAAAACGACGGCCAGT and M13r 5′-CAGGAAACAGCTATGAC were included at 5′ and 3′ ends of the LCS primers [24]. Each PCR mixture (final volume, 50 µl) contained 100 ng of template DNA, 0.1 µM of each primer, 0.50 mmol l^−1^ of each deoxynucleoside triphosphate, 2.5 mmol l^−1^ MgCl_2_, 5 µl of 10 X PCR buffer and 2.5 U of native Taq polymerase (Invitrogen, Milano, Italy). The following PCR conditions were used: 94°C for 5 min, followed by 20 cycles at 94°C for 1 min, 45°C for 1 min, 72°C for 2 min. A final extension was carried out at 72°C for 7 min. After agarose gel electrophoresis, PCR products were purified with a QIAquick gel extraction kit (Qiagen, Milano, Italy) and 20 ng of the purified product were used as template in a second PCR where primers M13f and M13r were used, with the addition of 454-adaptors and a 10 bp sample-specific Multiplex Identifier (MID). The PCR mixture was prepared as above described and the PCR conditions were the same, except for the annealing temperature, that was increased to 50°C. PCR products were purified twice by Agencourt AMPure kit (Beckman Coulter, Milano, Italy) and then quantified using the QuantiFluor™ (Promega, Milano, Italy). An equimolar pool of amplicons was prepared and it was used for pyrosequencing on a GS Junior platform (454 Life Sciences, Roche, Italy) according to the manufacturer’s instructions by using a Titanium chemistry and a bidirectional sequencing.

### Bioinformatics and Data Analysis

Raw reads were first filtered according to the 454 amplicon processing pipeline. Sequences were then analyzed by using QIIME 1.6.0 software [25]. Raw reads were demultiplexed and further filtered through the split_library.py script of QIIME. For *lacS* gene reads, the script was carried out twice, in order to demultiplex both forward and reverse reads, after obtaining the reverse complement. In order to guarantee a higher level of accuracy, the reads were excluded from the analysis if they had an average quality score lower than 25, if there were ambiguous base calls, if there were primer mismatches and if they were shorter than 300 and 200 bp, for 16S rRNA and *lacS* gene reads, respectively. Two different pipelines were used to analyse 16S rRNA and *lacS* gene reads. For 16S rRNA gene reads, the analysis was carried out as follows: sequences that passed the quality filter were denoised [26] and singletons were excluded. OTUs defined by a 97% of similarity were picked using the uclust method [27] and the representative sequences, chosen as the most abundant in each cluster, were submitted to the RDPII classifier [28] to obtain the taxonomy assignment and the relative abundance of each OTU using the Greengenes 16S rRNA gene database [29]. Alpha and beta diversity were evaluated through QIIME as recently described [30]. The OTU table filtered at 0.1% abundance was used to generate an OTU network by QIIME and a bipartite graph was constructed in which each node represented either a sample or a bacterial OTU. Connections were drawn between samples and OTUs, with edge weights defined as the number of sequences from each OTU that occurred in each sample. Network was visualized using Cytoscape 2.5.2 [31]. Moreover, OTUs tables generated through QIIME were used to draw a pseudo-heatmap in R environment (http://www.r-project.org) using gplots package. Representative sequences belonging to clusters identified as *Lactobacillus* spp. were double-checked using the BLAST (BLASTN) search program (http://www.ncbi.nlm.nih.gov/blast/).

Weighted UniFrac distance matrix were used to perform Adonis and Anosim statistical tests through the compare_category.py script of QIIME, in order to verify if there were differences among the three types of cheese. Moreover, the otu_category_significance.py script was run in order to test whether the presence/abundance of any OTUs was significantly associated to a specific cheese.

For the *lacS* gene reads, the analysis pipeline carried out was the following: forward and reverse-complemented demultiplexed sequences that passed the quality filters were denoised [26], using a sequence similarity threshold of 99%. After primer truncation, singletons were excluded and *lacS* gene sequence types defined by a 100% of similarity were picked using the uclust method [27]. The longest sequence of each cluster was picked as representative sequence. The representative sequences were aligned to the *lacS* sequence of the strain A147 (accession no. M23009) by using MEGA 5.2.2 software [32], manually checked in order to confirm mutations detected by QIIME and corrected. After alignment, a phylogenetic tree was built using the UPGMA (Unweighted Pair Group Method with Arithmetic Mean) method. Those clusters represented by sequences characterized by a 100% of similarity were merged and *lacS* sequence types were defined as having at least one point of mutation compared to the reference sequence.

### Nucleotide Sequence Accession Number

All the sequencing data were deposited at the Sequence Read Archive of the National Center for Biotechnology Information (SRP033419).

## Results

### 16S rRNA Gene Pyrosequencing

A total of 296,385 raw reads were obtained after the 454 processing; 221,903 reads passed the filters applied through QIIME, with an average value of 4,191 reads/sample and an average length of 469 bp. The number of reads obtained for each sample, the number of OTUs, the Good’s estimated sample coverage (ESC), the Chao1 and the Shannon indices are reported in Table S1. Rarefaction analysis showed that there was a satisfactory coverage for all the samples (ESC above 99% for all the samples).

**Figure 1:**
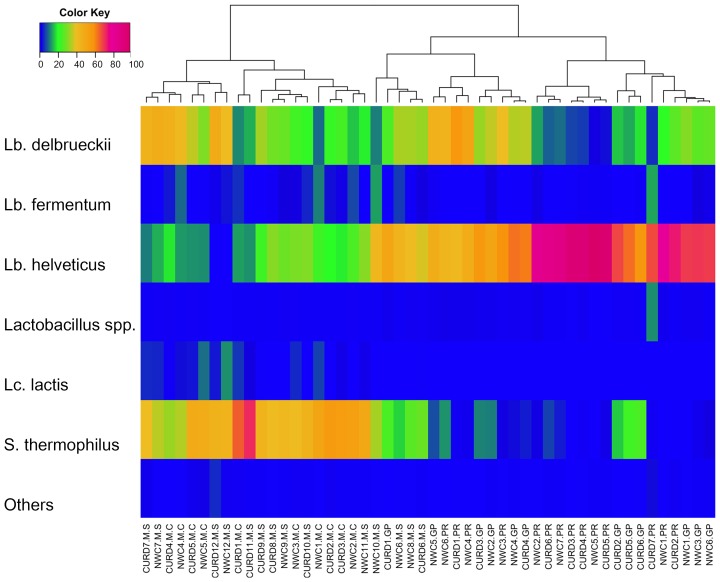
Pseudo-heatmap depicting distribution (%) of bacterial genera and species in NWC and curd samples from Grana Padano (GP), Parmigiano Reggiano (PR) and Mozzarella manufactures from Caserta (MC) and Salerno (MS) area of production. Only OTUs occurring at >1% abundance in at least 2 samples were included. Clustering of samples was obtained using Euclidean distance mesure and the average linkage method.

After QIIME analysis, 82 OTUs were identified, but only 6 had a relative abundance higher than 1% in at least two samples (Figure 1). *Lb. delbrueckii* and *Lb. helveticus* were the major OTUs in GP and PR samples (reaching a relative abundance of 59 and 93% of the total OTUs, respectively). *S. thermophilus* was also always present, but its abundance was never above 24%, with a higher abundance in GP samples. *Lb. fermentum* occurred only in some samples, but at very low percentage. On the contrary, samples from M manufactures were characterized by abundance of *S. thermophilus* (up to 70% of the total OTUs), while *Lb. delbruekii* and *Lb. helveticus* were present at lower extent compared to GP and PR samples (Figure 1). *Lactococcus lactis* and *Lb. fermentum* were also among the most represented OTUs in M samples, with a maximum abundance of about 13 and 12%, respectively (Figure 1). Many samples contained a low percentage of *Lactobacillus* sp., that was not possible to identify at species level. Representative sequences belonging to this cluster were double-checked using the BLAST (BLASTN) search program (http://www.ncbi.nlm.nih.gov/blast/). Although the identity scores were quite low, they were identified as *Lb. acidophilus* (87–95%), *Lb. johnsonii* (90–93%), *Lb. gasseri* (92–94%), *Lb. crispatus* (86–95%).

**Figure 2:**
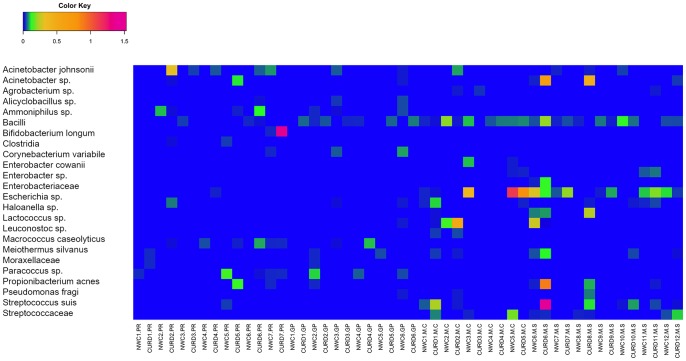
Pseudo-heatmap depicting distribution (%) of bacterial genera and species in NWC and curd samples from Grana Padano (GP), Parmigiano Reggiano (PR) and Mozzarella manufactures from Caserta (MC) and Salerno (MS) area of production. Only OTUs (except those reported in Fig. 1) occurring at >0.01% abundance in at least 2 samples were included.

Moreover, sub-dominant populations were also identified and 25 OTUs occurred with an abundance higher than 0.01% in at least 2 samples (Figure 2). M samples showed a higher complexity and many sub-dominant species reached abundandances higher than GP/PR samples. Many of them belonged to *Enterobacteriaceae* family or to the LAB group (*Lactococcus* sp. and *Leuconostoc* sp.). *Acinetobacter johnsonii* and *Acinetobacter* sp. reached 0.5% in some curd samples from all the three different manufactures. *Propionibacterium acnes* was found only in GP and PR samples (0.02–0.07%), while *S. suis* was present only in M samples (0.02–0.2%). *Bifidobacterium longum* occurred only in one PR NWC and in the relative curd.

**Figure 3:**
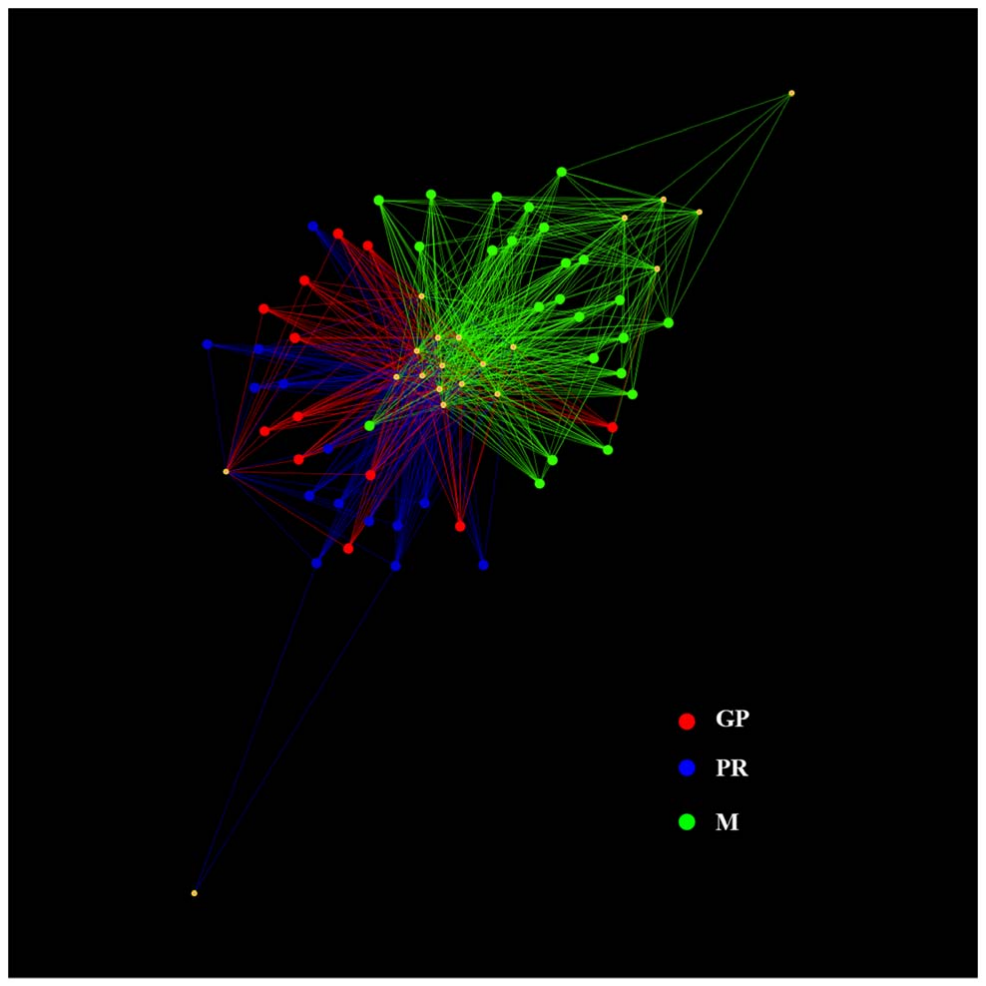
Simplified illustration of possible cheese - microbe networks. Network nodes are color coded by cheese type. Only OTUs with abundance >0.1% were considered.

The OTU network in Figure 3 showed clearly that samples from M manufactures clustered separately from GP and PR samples. However, a core microbiota of few OTUs was shared among the samples (*S. thermophilus*, *Lb. delbrueckii*, *Lb. helveticus*, *Lb. fermentum*, *Lactobacillus* sp., and some sub-dominant OTUs previously discussed) while some M or GP/PR specific OTUs can be also identified. The same conclusion was drawn from β-diversity analysis (data not shown). The statistical Adonis and Anosim tests run by QIIME showed that the samples significantly differed (P<0.001) according to cheese type. Moreover, the ANOVA and g test run through the otu_category_significance.py script of QIIME showed that *S. thermophilus*, *Lc. lactis* and *Lb. helveticus* abundance was significantly different in the three cheeses (P<0.001). On the contrary, no significant difference was found between M samples from Salerno (MS) and Caserta (MC) area (P>0.05).

### *lacS* Gene Pyrosequencing

**Figure 4:**
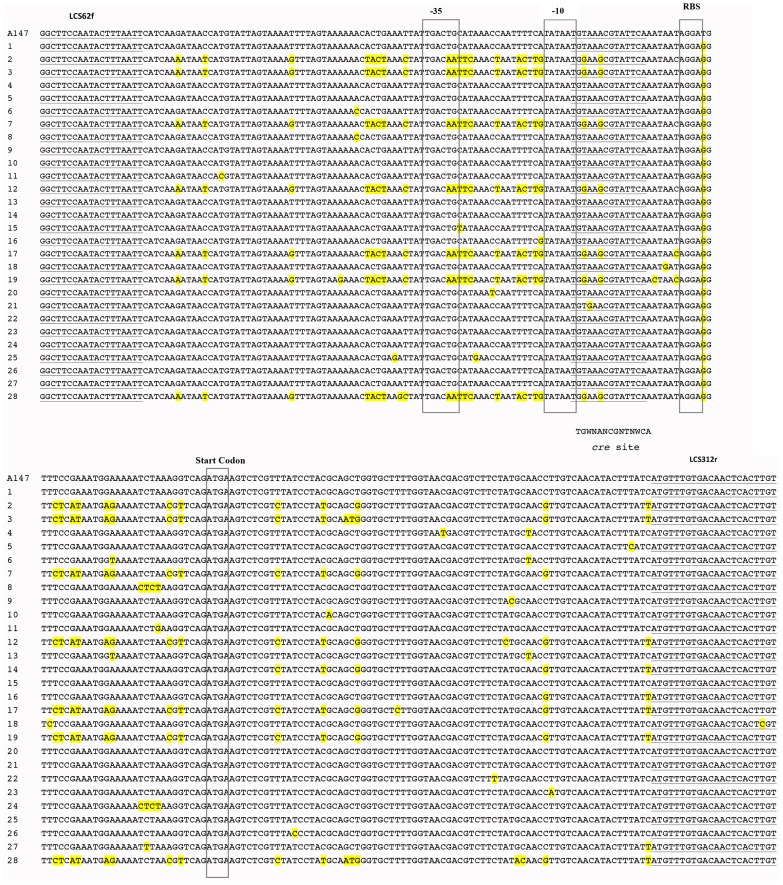
Nucleotide sequence alignment of the 28 *lacS* gene sequence types identified in this study. Sequences are aligned to the reference sequence of strain A147 (accession no. M23009). The ribosome binding site (RBS), the −35 and −10 sequences and the start codon are boxed. The putative *cre* site is underlined and aligned with the consensus sequence. The primer sequences are underlined.

**Figure 5:**
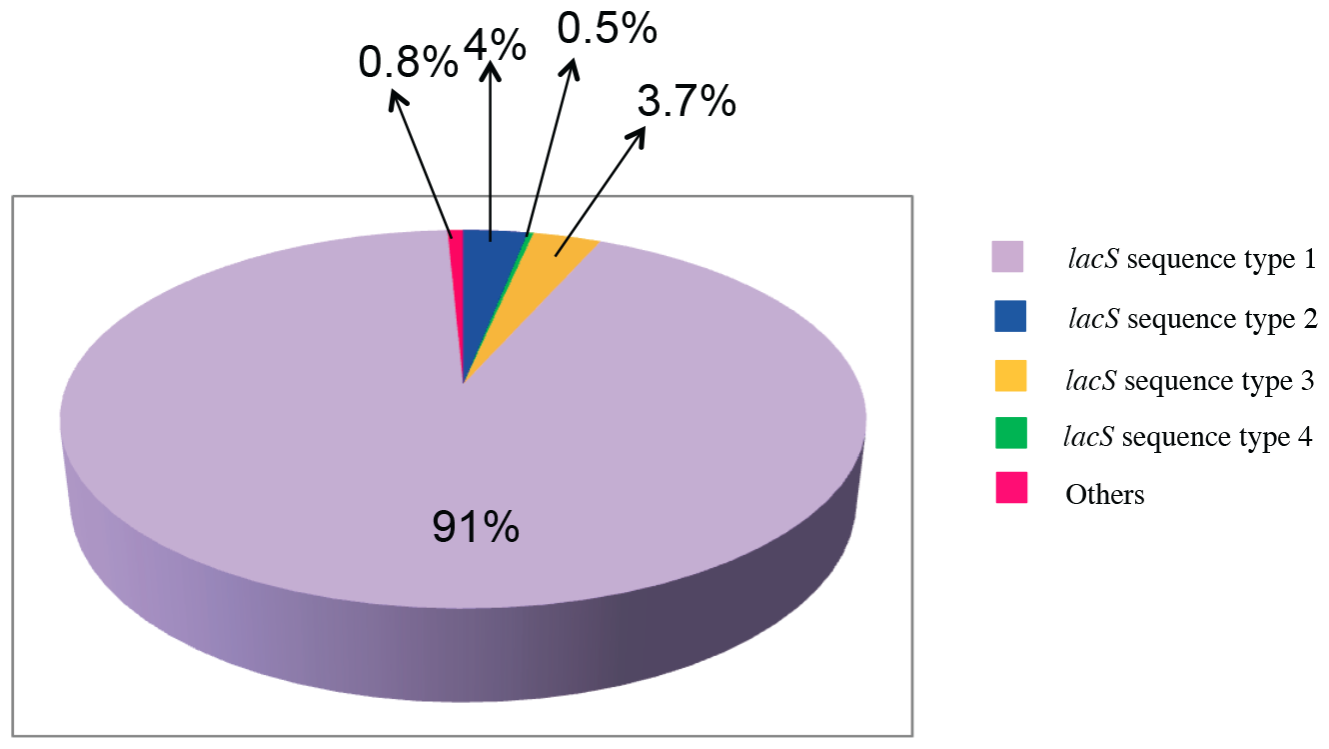
Abundance (%) of the *lacS* sequence types identified in this study. The abundance values are averaged for the three cheeses and only sequence types with an occurrence of at least 0.5% are showed in the color legend.

The results of 16S rRNA gene sequencing showed that *S. thermophilus* abundance was different in the three cheese types. A further analysis was carried out in order to investigate *S. thermophilus* variability at biotype level, through pyrosequencing of *lacS* gene amplicons. A total of 226,008 raw reads were obtained from *lacS* gene pyrosequencing; 195,315 passed the filters applied through QIIME, with an average value of 3,488 reads/sample and an average length of 250 bp. Clustering at 100% of similarity allowed identification of 28 different sequence types, but only 13 of them had a relative abundance higher than 1% in at least one sample. The average percentage of the 6 most abundant *lacS* types in the three manufactures is reported in Table 1. In particular, MC samples showed the highest number of different *lacS* types, while GP the lowest. The mutations identified are reported in Figure 4, while the abundance of the *lacS* types is shown in Figure 5. One *lacS* type (*lacS* type 1) occurred very often, with abundance ranging 87–99% in almost all the samples. A total of 60 mutation points could be detected that allowed the differentiation of 28 *lacS* gene sequences. Most of the differences were found in the promoter region upstream from the *lacS* gene. Within the promoter region, −10 regions did not show sequence variability, whereas region −35 turned from TTGACT to TTGACA in 9 out of 28 reference sequences. Twenty-seven points of mutations were found in the protein coding sequence of the gene (Figure 3), but only some of them led to amino acid changes in the primary structure of the protein. In particular, only 9 amino acid changes were found in the putative protein. A dendrogram of similarity of the 28 different sequence types identified is reported in Figure S1 where 2 major sequences clusters can be identified.

**Table 1:** Total number and average relative abundance (%) of the prevalent *lacS* sequence types identified in NWC and curd samples from Grana Padano (GP), Parmigiano Reggiano (PR) and Mozzarella manufactures from Caserta (MC) and Salerno (MS) area of production.

Sequencetype	GP	PR	MC	MS
1	98	98.7	69	99.5
2	–	–	14	–
3	–	<0.5	15	<0.5
4	1	<0.5	–	–
5	<0.5	0.5	–	–
6	<0.5	0.5	–	–
Total n° ofsequence typesidentified	9	16	19	10

Only types occurring at >0.5% abundance were included.

## Discussion

In this study, the microbiota of NWC and curds from manufactures of three traditional Italian cheeses was analyzed. Culture independent HTS was used for an in depth quantitative determination of the structure of the microbial populations.

NWCs are generally characterized by a relatively simple LAB microbiota. This LAB community is generally thermophilic and well adapted to the peculiar physico-chemical conditions (e.g. low pH and redox) of the whey substrate [33], [34]. Therefore, methods allowing direct DNA analysis from these environments are valuable in order to avoid biases of the culture-dependent approach. 16S rRNA pyrosequencing has been recently applied to analysis of microbiota in dairy matrices [3], [35]–[37] and it is regarded as an extremely sensitive tool, able to reveal also sub-dominant OTUs [38].

16S rRNA sequencing revealed a very simple microbial community in all the types of cheese. GP and PR cheeses were characterized by a very similar microbiota and curd fermentation seemed to be driven by *Lb. delbrueckii* and *Lb. helveticus*. Accordingly, *Lb. helveticus* and *Lb. delbruekii* were reported as dominant and *Lb. fermentum* and *S. thermophilus* as sub-dominant species in NWCs for GP manufactures [10]. *S. thermophilus* was generally present at very low concentration in NWCs for PR, reaching the 12% only in one sample. Moreover, it did not increase in abundance during curd fermentations, suggesting a minor contribution to acidification. On the contrary, GP curd ripening conditions seemed to be more suitable to the development of this microorganism, since a higher amount of *S. thermophilus* was found in all the curds, compared to the corresponding NWCs. This was more likely related to the curd ripening conditions, rather than to the abundance of this OTU in the NWC. In fact, a higher abundance of *S. thermophilus* in the NWC, did not lead to a higher amount of this OTU in the relative curd (Figure 1). In agreement with our results, *S. thermophilus* was found more frequently in GP compared to PR NWCs [39]. Higher abundance of *S. thermophilus* in GP curds could be due to a lower cooking temperature (51–48°C vs 53–54°C). A thermal gradient that starts with a lower temperature can cause a reduction of the heat stress within the molded curd, favoring the presence of *S. thermophilus* [39]. For the same reason, the mesophilic *Lb. fermentum* was more often found in GP than PR. Accordingly, *Lb. fermentum* was even more abundant in M since no curd cooking is employed in mozzarella cheese production.

As shown in Figure 1, many samples contained a low percentage of *Lactobacillus* sp., that was not possible to identify at species level, and that possibly belonged to non-starter LAB (NSLAB) group. NSLAB are often isolated from whey cultures [1], [40]; they do not contribute to acid production during manufacture, but can play a significant role during ripening [4], [39]. Mozzarella whey starters were characterized by a higher abundance of *S. thermophilus*, that often increased in abundance during curd ripening, together with thermophilic lactobacilli. *Lc. lactis* and *Lb. fermentum* were present at lower concentration and not in all the samples. In particular, *Lc. lactis* was present only in M samples, even if not in all the manufactures (Fig. 1) and its presence was significantly correlated to M samples, as confirmed by the g test (P<0.001). This microorganism was previously suggested to be related to the lower level of industrialization of the manufacture, correlating the occurrence of this species in traditional dairy products obtained from unselected microbiota and non-pasteurized milk [41]. Even if raw milk used in these manufactures was not analysed, the same OTUs were found both in the NWC and in the relative curd, indicating that the fermentation is driven by the NWC and that the microorganisms present in the milk do not play a key role [3]. The OTU network in Figure 3 clearly showed a separation between M and PR/GP samples. A few abundant OTUs constituted a shared core microbiota between the three cheeses. Although they are completely different cheeses, the fermentation process is most probably entirely relying on those common species. Also 25 sub-dominant OTUs were identified (Fig. 2). M samples showed a higher complexity, likely due to a less industrialized manufacture. Many sub-dominant OTUs are clearly environmental contaminants, like *Escherichia* sp., *Enterobacter cowanii* and other OTUs belonging to *Enterobacteriaceae* family. *Agrobacterium* sp. and *Alicyclobacillus* sp. probably arose from soil and agricultural environment [42], [43]. On the contrary, *Propionibacterium acnes*, found only in GP and PR samples, was likely of human origin [44]. *Pseudomonas fragi* was often associated to milk and dairy products [3], [45], where is able to produce volatile esters [46]. *S. suis*, an emerging zoonotic pathogen that can be transmitted to human [47], was found only in M samples. It was previously found in Mozzarella [3], as well as in another pasta-filata cheese [48]. However, the distribution of these low-abundance OTUs was really variable among the samples, suggesting that their presence is associated to sporadical contaminations. As previously suggested [38], [49], a RNA-based approach would be useful to understand which of these OTUs are metabolically active.

Since *S. thermophilus* is a very important species for the dairy industry and it was present in all the three manufactures, a further analysis was carried out in order to explore the biodiversity occurring within *S. thermophilus* species. It was often found in the NWC of several traditional cheeses [3]–[5],[8],[16]. Its genome is shaped by its domestication to the dairy environment, with gene features that conferred rapid growth in milk, stress response mechanisms and host defense systems that are relevant to its industrial applications [50]. Since many studies highlighted the presence of strain-specific phenotypic traits, such as exopolysaccharide production [51], urease activity [52], [53], galactose fermentation [54]–[56] and nitrogen metabolization [57], monitoring of this species at biotype level can be very interesting in order to trace biotypes with specific traits during cheese manufactures. Several strain-monitoring methods have been proposed, based both on phenotypic and genotypic approaches [19]–[22], [52], [55]. All these methods are based on cultivation of strains prior to molecular analysis, so they are subject to the well-known biases of culture-dependent methods. In fact, the presence of a high unculturable fraction of *S. thermophilus* in NWCs for GP production has been reported [58]. The occurrence of species-specific genes with substantial sequence heterogeneity can be a valid premise to apply high-throughput sequencing strategies to achieve strain specific monitoring without cultivation [38]. In this study, a high-thoughput sequencing of *S. thermophilus lacS* gene was carried out, in order to explore the biotype diversity in the samples studied and to evaluate the possibility of using this technique to monitor *S. thermophilus* biotypes without cultivation. Overall, the sensitivity of pyrosequencing allowed identifying some low-frequency mutations representing less than 1% of the total *lacS* sequences. Although we have no mean to be 100% sure that when only one mutation occurs this is not due to pyrosequencing error, we decided to define a *lacS* type as having at least one mutation point compared to the reference sequence of the strain A147. Our results showed that only few *lacS* types of *S. thermophilus* were abundant in all the samples (Fig. 4). In particular, one of them dominated in all GP and PR samples and in all M samples from Salerno area (MS). Such *lacS* type was also present in M samples from Caserta dairies (MC), which had the highest variability in *lacS* types, indicating a higher diversity of *S. thermophilus lacS* types in this area of production. In fact, *lacS* type 2 was present only in MC, while the type 3, although present also in some MS at very low concentration, was a dominant *lacS* type in MC. Sequence types 2 and 3 represented 14 and 15% of the total *lacS* sequences of MC samples, respectively (Table 1). In all the cases, the same *lacS* types were found both in the NWC and in the relative curd (data not shown). The dominance of *lacS* type 1 (Figure 5 and Table 1) that was the most abundant sequence type in all the samples is probably due to the insufficient level of heterogeneity of the *lacS* gene within *S. thermophilus* biotypes.

The lac operon in *S. thermophilus* contains the *lacZ* gene encoding for the β-galactosidase located downstream from the *lacS* gene that encodes for the lactose permease LacS, both controlled by the same promoter, located upstream from the *lacS* gene [59], [60]. A 250 bp variable region of the *lacS* gene was targeted, since it showed a high level of heterogeneity in a previous study [23]. Pyrosequencing allowed identification of a total of 60 mutation points, most of all in the region upstream from the *lacS* gene, in agreement with the previous study [23]. In particular, in 9 out of 28 sequences a mutation in the −35 box was found. This hexamer, beside to the −10 box, plays as a binding site for the δ subunit of RNA polymerase and allows the transcription to start [61]. Since the transcription level was not investigated, we do not know if this could be considered a down mutation, possibly affecting the transcription efficiency. The presence of a putative catabolite responsive element (*cre*) overlapping the −10 box was previously highlighted [23], indicating a possible role for regulation by a catabolite control protein A (CcpA) [59]. Our results confirmed that this is a conserved region, except for three points of mutation: a T and two A turning all to G at position 3, 4 and 6 of the *cre* site (underlined in Fig. 4), respectively. The mutations in position 3 and 6 were already pointed out by Ercolini et al. [23], while the one in position 4 was identified in this study, even if this *lacS* type occurred at low abundance and only in few samples. However, only the substitution in position 3 creates a mismatch in the *cre* sequence.

This study provided an in-depth description of the microbiota involved in curd fermentation in three popular Italian cheese productions. The results showed a high degree of homogeneity in the microbiota involved in the early stages of the three dairy manufactures, highlighting a naturally-selected core microbiome that is fundamental for the fermentation in these dairy preparations. Moreover, a HTS based approach for culture-independent typing of microbiota beyond the species in food was developed in order to have a tool for monitoring of biotypes of certain species during food production. Although *lacS* gene did not prove enough variable within *S. thermophilus* species to be used for quantitative strain monitoring, we highlighted the possibility of using non rRNA genomic amplicons for a culture-independent identification of types within species in food matrices.

## Supporting Information

Figure S1 **Dendrogram of similarity of the 28** ***lacS*** **sequence types identified in this study.**
(TIFF)

Table S1 Number of sequences analyzed, observed diversity and estimated sample coverage for 16S rRNA amplicons analyzed in this study.
(DOCX)
