# Supplementary material for: A Selected Core Microbiome Drives the Early Stages of Three Popular Italian Cheese Manufactures
Source: PLoS One. 2014 Feb 24;9(2):e89680. doi: 10.1371/journal.pone.0089680 (PMC3933672; doi:10.1371/journal.pone.0089680)
Supplement: Table S1 — Number of sequences analyzed, observed diversity and estimated sample coverage for 16S rRNA amplicons analyzed in this study. (DOCX) [file pone.0089680.s002.docx]

**Table 1.** Number of sequences analyzed, observed diversity and estimated sample coverage for 16S rRNA amplicons analyzed in this study.

| **Sample** | **Reads** | **OTUs** | **Chao1** | **Shannon** | **ESC (%)** |
| --- | --- | --- | --- | --- | --- |
| **NWC1-PR** | 3789 | 38 | 62,00 | 1,16 | 99,58 |
| **CURD1-PR** | 3747 | 48 | 69,00 | 1,53 | 99,44 |
| **NWC2-PR** | 5627 | 36 | 51,00 | 1,35 | 99,72 |
| **CURD2-PR** | 2726 | 19 | 26,50 | 0,47 | 99,63 |
| **NWC3-PR** | 3172 | 40 | 68,50 | 1,26 | 99,40 |
| **CURD3-PR** | 6132 | 40 | 66,25 | 1,04 | 99,66 |
| **NWC4-PR** | 4513 | 47 | 68,38 | 1,56 | 99,58 |
| **CURD4-PR** | 4197 | 49 | 91,17 | 0,80 | 99,45 |
| **NWC5-PR** | 3889 | 53 | 86,33 | 1,05 | 99,36 |
| **CURD5-PR** | 3750 | 73 | 124,67 | 2,12 | 99,17 |
| **NWC6-PR** | 3877 | 47 | 98,00 | 2,14 | 99,54 |
| **CURD6-PR** | 5766 | 63 | 92,55 | 1,21 | 99,55 |
| **NWC7-PR** | 2782 | 28 | 39,00 | 0,50 | 99,60 |
| **CURD7-PR** | 4444 | 45 | 73,50 | 0,69 | 99,57 |
| **NWC1-GP** | 2980 | 34 | 40,60 | 1,31 | 99,60 |
| **CURD1-GP** | 3587 | 43 | 63,00 | 2,17 | 99,55 |
| **NWC2-GP** | 3863 | 58 | 145,00 | 2,07 | 99,22 |
| **CURD2-GP** | 4240 | 48 | 52,13 | 1,81 | 99,72 |
| **NWC3-GP** | 6052 | 64 | 83,25 | 1,59 | 99,64 |
| **CURD3-GP** | 4084 | 48 | 61,91 | 1,96 | 99,56 |
| **NWC4-GP** | 3857 | 59 | 122,00 | 1,67 | 99,27 |
| **CURD4-GP** | 3156 | 40 | 85,33 | 1,69 | 99,46 |
| **NWC5-GP** | 2350 | 37 | 56,50 | 1,93 | 99,45 |
| **CURD5-GP** | 3612 | 40 | 55,00 | 1,87 | 99,58 |
| **NWC6-GP** | 4787 | 41 | 54,91 | 1,30 | 99,62 |
| **CURD6-GP** | 4539 | 39 | 44,50 | 1,88 | 99,74 |
| **NWC1-M-C** | 4041 | 53 | 74,11 | 2,51 | 99,51 |
| **CURD1-M-C** | 5760 | 69 | 101,50 | 2,23 | 99,55 |
| **NWC2-M-C** | 2079 | 31 | 46,60 | 2,10 | 99,37 |
| **CURD2-M-C** | 4806 | 54 | 84,00 | 2,09 | 99,56 |
| **NWC3-M-C** | 4211 | 51 | 60,10 | 2,53 | 99,67 |
| **CURD3-M-C** | 3241 | 40 | 80,00 | 2,14 | 99,51 |
| **NWC4-M-C** | 4307 | 56 | 96,63 | 2,42 | 99,40 |
| **CURD4-M-C** | 4581 | 50 | 125,00 | 2,20 | 99,45 |
| **NWC5-M-C** | 4523 | 61 | 100,43 | 2,60 | 99,47 |
| **CURD5-M-C** | 4211 | 49 | 99,60 | 2,17 | 99,45 |
| **NWC6-M-C** | 4450 | 46 | 55,00 | 2,36 | 99,80 |
| **CURD6-M-C** | 7600 | 82 | 117,10 | 2,06 | 99,64 |
| **NWC7-M-S** | 4198 | 42 | 99,75 | 2,11 | 99,48 |
| **CURD7-M-S** | 4598 | 55 | 109,38 | 2,17 | 99,35 |
| **NWC8-M-S** | 4295 | 45 | 66,11 | 2,17 | 99,53 |
| **CURD8-M-S** | 4544 | 55 | 136,25 | 2,25 | 99,43 |
| **NWC9-M-S** | 4651 | 44 | 90,00 | 2,04 | 99,48 |
| **CURD9-M-S** | 4678 | 45 | 95,00 | 2,07 | 99,47 |
| **NWC10-M-S** | 5424 | 55 | 69,62 | 2,22 | 99,63 |
| **CURD10-M-S** | 4913 | 55 | 91,91 | 2,04 | 99,41 |
| **NWC11-M-S** | 4079 | 50 | 71,38 | 2,43 | 99,53 |
| **CURD11-M-S** | 4850 | 58 | 95,80 | 2,19 | 99,42 |
| **NWC12-M-S** | 18679 | 63 | 76,33 | 2,12 | 99,91 |
| **CURD12-M-S** | 11888 | 70 | 82,67 | 1,81 | 99,83 |

Abbreviations: OTU, operational taxonomic unit; ESC, estimated sample coverage. Chao1, Shannon and ESC were calculated with Qiime at the 3% distance level.
